# Time-resolved coherent X-ray diffraction imaging of surface acoustic waves

**DOI:** 10.1107/S1600576714016896

**Published:** 2014-09-04

**Authors:** Jan-David Nicolas, Tobias Reusch, Markus Osterhoff, Michael Sprung, Florian J. R. Schülein, Hubert J. Krenner, Achim Wixforth, Tim Salditt

**Affiliations:** a Institut für Röntgenphysik, Friedrich-Hund-Platz 1, 37077 Göttingen, Germany; b Deutsches Elektronen-Synchrotron, Notkestrasse 85, 22605 Hamburg, Germany; c Lehrstuhl für Experimentalphysik I, Universitätsstrasse 1, 86159 Augsburg, Germany; d Nanosystems Initiative Munich (NIM), Schellingstrasse 4, 80799 München, Germany

**Keywords:** coherent X-ray diffraction imaging, surface acoustic waves, nanostructure

## Abstract

Time-resolved coherent X-ray diffraction experiments of standing surface acoustic waves, illuminated under grazing incidence by a nanofocused synchrotron beam, are reported. The data have been recorded in stroboscopic mode at controlled and varied phase between the acoustic frequency generator and the synchrotron bunch train. At each time delay (phase angle), the coherent far-field diffraction pattern in the small-angle regime is inverted by an iterative algorithm to yield the local instantaneous surface height profile along the optical axis. The results show that periodic nanoscale dynamics can be imaged at high temporal resolution in the range of 50 ps (pulse length).

## Abstract 2

The time-dependent one-dimensional height profile of a standing surface acoustic wave on an LiNbO_3_ substrate has been reconstructed from stroboscopically recorded coherent grazing-incidence small-angle diffraction patterns.

## 1. Introduction   

Coherent X-ray diffraction imaging (CXDI) has emerged as a powerful technique to analyze the nanoscale structure of materials (Robinson *et al.*, 1999 ▶; Miao *et al.*, 1999 ▶, 2012 ▶; Robinson & Harder, 2009 ▶ Vartanyants & Yefanov, 2013 ▶), compatible with a wide range of sample preparations and environmental parameters. This is of significant advantage in view of functional materials. A major persisting challenge in order to unravel functional mechanisms in materials and devices, however, requires an extension of static studies to time-resolved imaging and microscopy. While advanced free-electron laser sources offer ultimate time resolution in the femtosecond range (Chapman, 2009 ▶; Chapman *et al.*, 2011 ▶) needed, for example, to study the dynamics of electrons, synchrotron radiation (SR) sources are well suited to cover the picosecond and nanosecond time scales, as demonstrated by a wide range of time-resolved diffraction experiments (Schotte *et al.*, 2002 ▶; Helliwell & Rentzepis, 2007 ▶).

**Figure 1:**
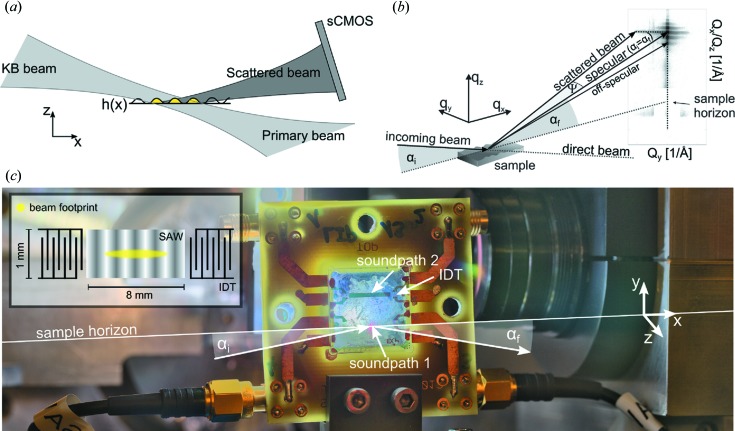
Schematics of the experimental geometry. (*a*) The illumination profile of a focused KB beam illuminating several SAW wavelengths (yellow region) on the substrate. (*b*) The coherent far-field diffraction pattern is collected at a distance of 5.2 m behind the sample. (*c*) In the experiment, the SAW device was placed vertically into the beam. Interdigital transducers (labeled IDT) define the SAW wavelength and frequency. After proper synchronization, the signal applied to the IDTs was varied in phase, amplitude and frequency.

**Figure 2:**
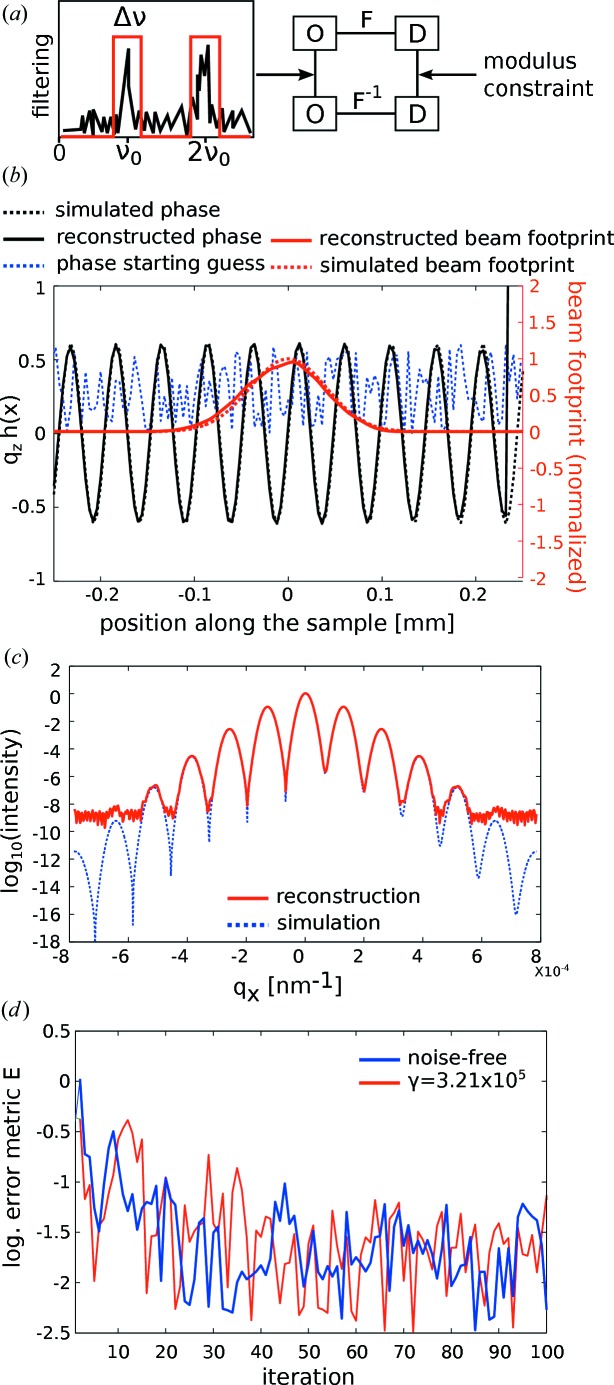
(*a*) Block diagram of the modified Gerchberg–Saxton algorithm with modulus and bandwidth constraint. O denotes the object and D the detector space. (*b*) Reconstructed relative phase φ and beam profile (normalized amplitude) along the sample from simulated data. The starting guess (blue, dotted) was taken to be constant but can also be a random guess. (*c*) Forward-simulated diffraction pattern, corresponding to the input data of the simulation, as calculated by a Fresnel–Kirchhoff integral, compared with the diffraction data of the final reconstruction. Even though the beam profile (illumination function) 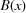 is unconstrained (except by reality), it is well reconstructed apart from minor distortions. The SAW pattern is well reconstructed in view of its magnitude, and the reconstructed profile is in exactly the right position with respect to the beam, *i.e.* its envelope function. (*d*) Logarithmic error metric 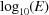 shown for two simulated data sets (noise-free and Poisson noise, expectation value 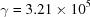).

**Figure 3:**
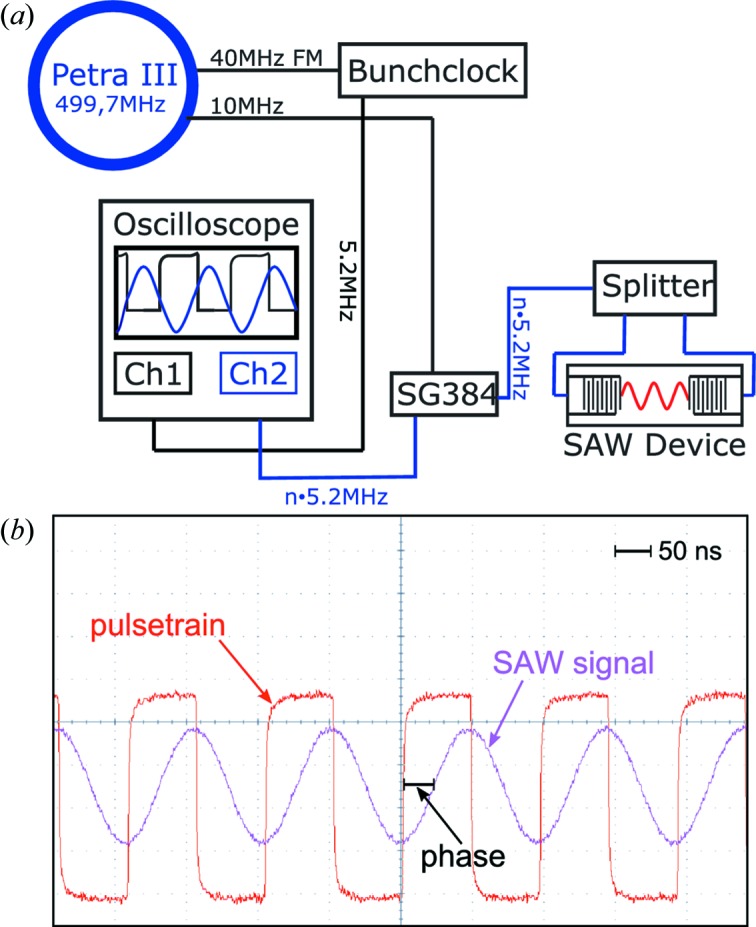
(*a*) Overview over the timing system used for phase-controlled time-resolved coherent X-ray diffraction. The Petra III bunchclock provides a signal corresponding to the respective bunch frequency (5.2 MHz in the 40-bunch mode) as well as a variety of preinstalled integral dividers. An accurate 10 MHz signal is used in order to lock a frequency generator (SG384) to the synchrotron frequency. (*b*) Oscilloscope trace as taken during phase-locked SAW generation, shown here for 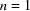. The rising edge of the red trace indicates the arrival of an electron bunch. Successive synchrotron pulses corresponding to the bunches (red) probe the standing SAW (pink) at a constant phase ϕ.

With this in mind, the scope of the present work is a first technical step in linking the experimental capabilities to perform time-resolved stroboscopic experiments at synchrotron sources with the imaging capability of coherent radiation. To this end, we have chosen surface acoustic waves (SAWs) on piezoelectric surfaces (Royer *et al.*, 1999 ▶; see the setup sketched in Fig.  1 ▶) as a well controlled periodic dynamical phenomenon which at the same time as being experimentally amenable is also of significant technological interest (Ballantine *et al.*, 1996 ▶; Länge *et al.*, 2008 ▶). As shown before in surface diffraction experiments, the radio frequency (RF) generator driving the SAW can be synchronized to the synchrotron pulses (Roshchupkin & Tucoulou, 1998 ▶; Sauer *et al.*, 1999*a* ▶,*b* ▶). Recently, the synchronization scheme was extended to allow for control and variation of the relative phase between the SAW and the radiation pulses (Reusch *et al.*, 2013 ▶). Here, we use coherent X-ray diffraction imaging in the surface-sensitive geometry of grazing incidence (Renaud, 2009 ▶) and phase retrieval, as shown in Fig. 2 ▶, in combination with this advanced timing scheme, which is summarized in Fig. 3 ▶. The time resolution achieved by this approach extends previous coherent X-ray diffraction experiments, as pioneered by, for example, Vartanyants *et al.* (1997 ▶) and Robinson *et al.* (1999 ▶), which have demonstrated reconstruction of static one-dimensional surface height functions, as well as more recent reconstructions of two-dimensional height functions (Yefanov *et al.*, 2009 ▶; Sun *et al.*, 2012 ▶).

This paper is organized as follows: §[Sec sec2]2 gives a brief conceptual background explaining the method of surface reconstruction as supported by computer simulations. All methods needed for this work (coherent nanofocusing, SAW devices, timing and synchronization scheme) are briefly described in §[Sec sec3]3. Experimental data are presented in §[Sec sec4]4, followed by the reconstruction results in §[Sec sec5]5, before the manuscript closes with a brief summary and outlook.

## 2. Reconstruction method and simulations   

The well developed framework of kinematical and semi-kinematical surface scattering theories (Sinha *et al.*, 1988 ▶; Tolan, 1999 ▶; Daillant & Gibaud, 1999 ▶), including more recent work on coherent surface diffraction (Tolan & Sinha, 1998 ▶; Madsen *et al.*, 2005 ▶; Gutt *et al.*, 2008 ▶), forms a solid basis for the present work. In the simplest approach, which is sufficient for the current purpose, we consider the coherent far-field scattering intensity distribution around the specular peak (including the specular peak), which is then used in combination with (real space) constraints to reconstruct the height profile 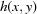. The corresponding surface structure factor describing the far-field diffraction amplitude can be written as
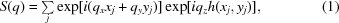
where 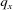, 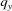 denote in-plane components and 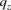 the out-of-plane (vertical) components of the momentum transfer. The sum is over all atoms in the illuminated area, and 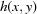 is the surface height function. In the simulations, coarse-grained scattering centers with separations much larger than atomic distances are used, since the low momentum transfer in reflectivity and grazing-incidence small-angle X-ray scattering justifies a continuum description. For coherent illumination, the measurable intensity is given by the modulus squared of the entire sum, 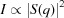, without further averaging over coherence volumes or stochastic realizations of the height function. The scattering at any time is a function of the instantaneous height profile corresponding to the SAW. The structure factor 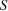 is thus given as the two-dimensional Fourier transform of a complex-valued function 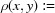 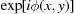:
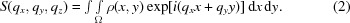



 The phase angle 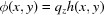 corresponds to the path length difference originating from the height difference between two reflection points, and Ω is the (illuminated) surface area. The idealized expression above implicitly assumes plane wave illumination yielding an amplitude one. However, in view of the experimental situation we have to consider the real-valued amplitude 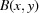 describing the beam profile on the sample surface. In general, 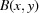 can be complex valued, for example, in the presence of wavefront curvature or aberrations of the incident beam. For the present experiment where the sample is placed in the focal plane of the Kirkpatrick–Baez (KB) mirror optics, it is reasonable to assume planar wavefronts, however, and to therefore impose reality of 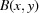. The quantity to be reconstructed in the coherent surface experiment then is 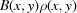.

**Figure 4:**
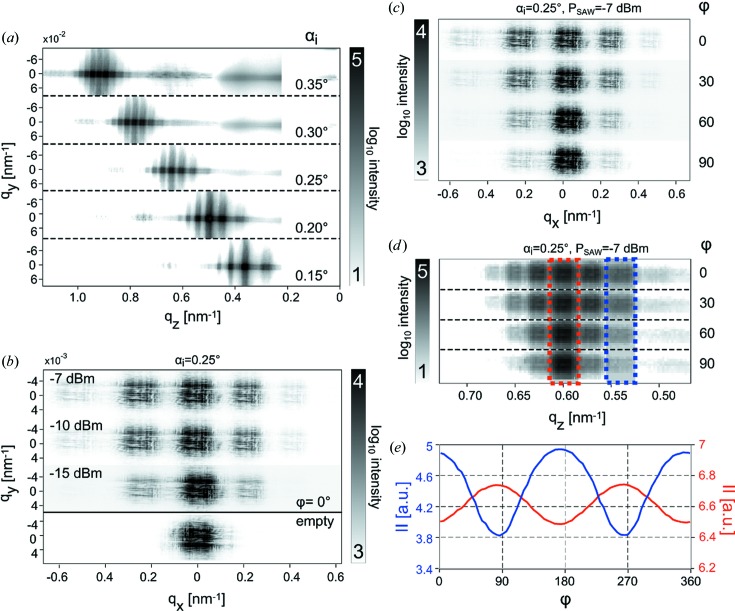
Coherent specular and nonspecular reflectivity signal, as recorded for (*a*) the Pilatus and (*b*) the sCMOS detector, for (*a*) different values of 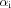 and (*b*) different amplifications. The horizontal direction (on the detector) corresponds to the 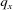 variation and the vertical direction to 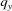. The position and spacing of the satellite peaks corresponds to 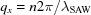. The variation of the signal with 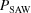 and the absence of satellites for zero SAW power confirm the successful timing scheme. The pattern of the high-resolution diffraction peaks shown in (*b*) resembles the coherent far-field pattern of the KB beam. (*c*), (*d*) Coherent far-field scattering intensity distribution as measured by (*c*) the sCMOS and (*d*) the Pilatus detector, for four selected values of the phase ϕ of the SAW signal. The integrated intensity of the specular and second-order reflections [marked with, respectively, a red and blue bounding box in (*d*)] is displayed in (*e*) as a function of the SAW phase ϕ. The strict periodicity confirms successful phase locking.

Next, we apply further specifications and assumptions geared towards the present experiment. First we have to keep in mind that the illuminated area on the sample surface is highly anisotropic at glancing incidence angle 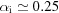
°. The long axis of the beam footprint corresponding to the projection of the KB beam with a width 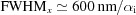 is directed along the sound path 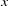 of the SAW, while the short axis in the vertical direction with a width of 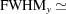 450 nm is perpendicular to the sound path. Since the acoustic wavefront of the SAW is sufficiently smooth we can neglect any variations along 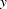 over the illuminated area and consider the height profile 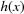 as a function of only one spatial dimension, *i.e.* the SAW propagation axis (sound path). By integration of the intensity along 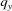 (the vertical direction on the pixel detector in Fig. 4 below), the experimental data are also reduced in dimensions. At the same time the height varies on the nanosecond time scale, so the final problem is to reconstruct a time-varying height profile illuminated stroboscopically by a stationary (but unknown) beam profile, *i.e.* 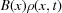.

In the far-field limit we record the modulus squared of the Fourier transform, 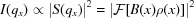. The missing phase needed for direct reconstruction of the object function is retrieved from additional constraints on the solution, using an iterative sequence of projectors, as well known from the literature (Fienup, 1982 ▶; Elser, 2003 ▶; Marchesini, 2007 ▶). The diffraction amplitude is replaced in each iteration by the square root of the measured intensity. This is denoted as the modulus or data constraint, *i.e.* the constraint that the measured data impose on the solution. This modulus constraint is thus described by an operator 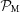, which replaces the amplitudes of the current wave function iterate in Fourier space 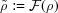, where 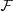 denotes the (one-dimensional) Fourier transform, by the square root of the measured intensities 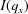:
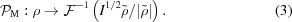



The second constraint is formulated in real space. Since the SAW fills the entire illuminated field of view, the standard constraint of a compact support, applicable for the error reduction or hybrid input–output algorithms (Fienup, 1982 ▶), cannot be used. A modulus constraint in real space projecting ρ onto a set of wave functions with known amplitude, such as in the Gerchberg–Saxton (GS) algorithm (Gerchberg, 1972 ▶), is also not applicable since the illumination function 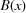 is unknown. However, with *a priori* knowledge about the SAW-induced height profile, in particular regarding the expected wavelength of the SAW (and the corresponding bandwidth of the signal), it is reasonable to assume a corresponding smoothness of 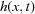. This is implemented by calculating the discrete Fourier transform in real space and then filtering the signal with a certain bandwidth 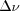 at central frequencies which are higher harmonics of the fundamental SAW frequency 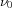. In the following, we will denote this constraint as the bandwidth constraint.

The iterative procedure is depicted in Fig. 2 ▶ along with a successful reconstruction based on a simulated data set. After proper initialization, the algorithm iterates between real space (denoted by O for object) and reciprocal space (denoted by D for detector), applying the modulus constraint in reciprocal and the bandwidth constraint in real space. The error of the reconstruction is monitored through the functional 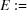 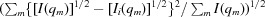. Inherent to this formulation is the assumption of a constant 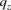. However, by measuring reciprocal space with a pixel detector at constant 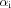, 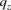 becomes a function of 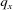. Because of the small 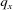 range probed, we neglect this dependence and take the average value 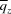. With this assumption in mind we can summarize the problem of one-dimensional iterative surface height reconstruction by the Fourier pair of equations (omitting real-space filtering)
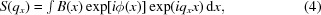


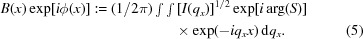



Given *a priori* knowledge about the illumination function in the focal plane, one can make an appropriate guess about the general shape of the wavefront 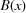, or even constrain the shape of 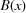 in all iterations. This can help to yield better convergence or to stabilize the algorithm. However, detailed knowledge about the illumination function 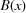 does not need to be provided in all cases to yield good reconstruction results.

In all reconstructions of this work, for both simulated and experimental data, we will, however, impose the reality of 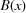, a condition warranted by the fact that the sample is in the focal plane of the KB mirrors. Before turning to the simulations that have been carried out in order to shed light on the reconstruction algorithms and the minimum set of constraints, there are a few general points that have to be considered when reconstructing a one-dimensional object function based on equations (4) and (5).

(*a*) Sampling: Given a discrete set of data points as measured by a pixel detector, equations (4) and (5) can be calculated using the fast Fourier transform (Cooley & Tukey, 1965 ▶) method. The sampling points of the diffraction pattern at locations 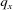 separated by a sampling interval 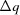 are related to the real-space sampling interval 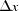 through the reciprocity relations of the Fourier transform (Goodman, 2005 ▶),
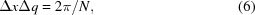
where 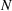 denotes the number of sampling points. In the experiment, 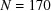 pixels are used, and a detector resolution 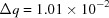
 µm^−1^ (as calculated from the detector pixel size), corresponding to a sampling interval of 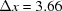
 µm along the surface.

(*b*) Uniqueness: A one-dimensional iterative algorithm of GS type as given by equations (4) and (5) has the disadvantage that infinitely many solutions for the phase 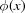 exist, *i.e.* it is generally not solvable uniquely (Paganin, 2006 ▶). Inverting a single diffraction pattern iteratively in one dimension is therefore only possible by including *a priori* knowledge about the function 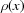. In this work, we use the mentioned bandwidth constraint to yield a unique solution, in addition to the modulus (data) constraint. Degeneracy of the solution can also be introduced through phase wrapping. Phase values outside the interval 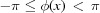 are wrapped to a value modulo 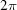. In our work, phase wrapping does not impose a problem since the phase φ is always included within this range. In fact, by control of 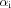, the range of phase values 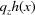 can be controlled. Of course, higher angles of incidence 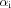 and height amplitudes may in general induce phase wrapping problems. However, even in these cases the bandwidth constraint employed in this work is able to suppress phase discontinuities, as confirmed by reconstructing a surface height profile with phase differences of 5 rad.

Note that the reduction to a spatially one-dimensional height profile and the corresponding one-dimensional reconstruction problem is a choice made in view of simplification. Indeed, extensions of this work towards reconstructions in two or three dimensions are attractive future directions. However, in this work the range of parallel momentum transfer covered in grazing incidence was highly anisotropic, which leads to an extremely anisotropic sampling of the 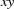 plane. Furthermore, for three-dimensional reconstruction the range of 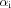 values was insufficient. We have therefore adopted the described approach of reducing the dimensions.

Before treating experimental data, simulations were performed to test the algorithm. The simulation parameters were chosen in view of the experimental parameters. In the results shown below, the SAW wavelength and amplitude were 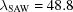
 µm and 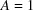
 nm, respectively. The incident angle was 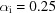
°, corresponding to an average vertical momentum transfer 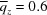
 nm^−1^. The photon energy was 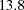
 keV. The central spatial frequency of the SAW is 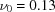
 µm^−1^ . The fundamental 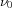 and the second and third harmonics were used for real-space filtering. The bandwidth was chosen to be 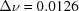
 µm^−1^. The beam profile was simulated with a full width at half-maximum 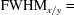 450 nm.

Fig. 2 ▶ illustrates the result of a reconstruction converging after 100 iterations. Fig. 2 ▶(*b*) shows the (unit-less) height profile 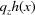 prior to and after reconstruction as well as the footprint of the elongated beam profile. The positions of the SAW maxima and minima within the beam envelope are faithfully reconstructed. Fig. 2 ▶(*c*) shows the excellent agreement of the reconstructed diffraction pattern (in the last iteration before application of the modulus constraint) compared to the input data of the simulation.

Finally, two reconstructions from a noise-free and a noisy data set (Poisson noise, expectation value 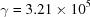) have been compared. In both cases the algorithm converges as monitored through the logarithmic error metric 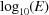 displayed in Fig. 2 ▶(*d*). Thus we can conclude that the algorithm described above is in principle able to reconstruct data of the type collected in this experiment.

## 3. Methods   

The experiment is based on a combination of SAW devices, a timing (synchronization) scheme between the synchrotron radiation pulses and the SAW dynamics, and the coherent nanofocus setup. Each part will be discussed separately in the following subsections. All parameters relevant to this experiment are summarized in Table 1 ▶, and further details are given in Appendix *A* [App appa].

### 3.1. Surface acoustic waves   

The propagation of waves in the 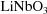 substrate is described by the wave equation for piezoelectric materials (Royer *et al.*, 1999 ▶). The waves excited here correspond to solutions of the Rayleigh type, *i.e.* surface waves with elliptical motion of lattice atoms in a plane given by the surface normal and the propagation direction. Standing SAWs of Rayleigh type are generated by two interdigital transducers (IDTs) placed at opposing ends of the acoustically active region (sound path) on the substrate, and matched in frequency and phase. The sound path in between the two IDTs is 8 mm long and 1 mm wide (see Fig. 1 ▶). The SAW wavelength is given by the spacing of the comb-like structure of the IDTs. The SAW amplitude can be tuned by an amplification of the signal power, applied to the transducers. To excite a SAW of Rayleigh type, different crystal cuts can be chosen, corresponding to different diffraction planes. In the present case, the commonly used 128° rot. Y cut [corresponding to (104) orientation] was used.

### 3.2. Timing   

Data acquisition is based on the pump–probe scheme. The periodic SAW excitation (pump) is synchronized to the PETRA III (probe) synchrotron radiation pulses. The coherent diffraction pattern is then accumulated by repeated probe pulses at constant time delay 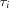 (*i* = 1,…, *N*) (stroboscopic illumination). For the 40-bunch filling mode, a bunch repetition rate of 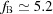
 MHz (determined by circumference, microwave frequency and electron energy) sets the timing for probing the structure. The frequency of the SAW 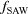 is then adjusted to an integral multiple of the bunch frequency. In view of the weak diffraction signal, flux limitations are circumvented by data accumulation at these high repetition rates. In order to vary τ, it is necessary to phase lock the frequency generator to the bunch frequency, which is achieved through a 10 MHz reference signal, derived from the microwave frequency using an integral divider and distributed to the beamlines at PETRA III (Reusch *et al.*, 2013 ▶, 2014 ▶). Variation of the phase is then used to probe the structure at time delay 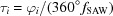. Fig. 3 ▶ summarizes the timing scheme implemented at beamline P10. To clarify the notation, we denote the phase for shifting the SAW signal with respect to the SR pulses by ϕ, while the phase of the exit wave (in the focal plane) is denoted by φ.

### 3.3. Nanofocus setup   

The experiment was carried out at P10 at the synchrotron facility PETRA III at DESY Photon Science (Hamburg, Germany), using the coherent nanofocus endstation GINIX (Göttingen Instrument for Nano-Imaging with X-rays) (Kalbfleisch *et al.*, 2010 ▶; Salditt *et al.*, 2011 ▶), with 13.8 keV photon energy selected by the Si(111) monochromator. At a spectral resolution of 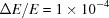, the requirement for longitudinal coherence is safely exceeded. The beam was focused onto a focal spot of 450 × 600 nm in the vertical and horizontal directions using two KB mirrors as focusing device, with entrance slits in front of the KB vacuum chamber closed to full spatial coherence, *i.e.* 50 µm in the horizontal and 75 µm in the vertical direction. The sample was positioned into the focus of the KB beam and the zero angle was calibrated by recording the specularly reflected beam at various angles of incidence 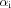. Primary radiation that is transmitted through the sample was blocked using a beam stop to protect the detector. The beam footprint in the propagation direction on the SAW device was roughly 500 µm, leading to local illumination of a few SAW wavelengths. Coherent diffraction patterns of the specular and nonspecular reflectivity were recorded using two different area detectors placed 5.2 m downstream from the sample. A Pilatus 300K detector (Dectris, Switzerland) with 172 µm pixel size was used to measure the coherent diffraction pattern with high dynamic range and at single-photon sensitivity, while a fibre-coupled scintillator sCMOS camera (Photonic Science) with a pixel size of 6.75 µm was used to sample the far-field pattern at higher spatial resolution.

## 4. Experimental results   

Three types of scans have been carried out:

(1) A rocking (angular) scan in the range 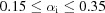
° with 0.05° incremental steps, as shown in Fig. 4 ▶(*a*) for data collected with the Pilatus pixel detector. The spacing of the satellite peaks increases with decreasing angle, as expected from the conversion of 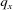 to exit angle 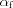.

(2) An RF power scan in the range of −23 to −7 dBm for all angles 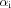 together with the corresponding reference diffraction patterns at zero SAW power. Fig. 4 ▶(*b*) shows examples of data at different SAW powers 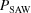, as measured with the sCMOS detector. A larger 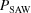 clearly leads to an increase in intensity of the satellite peaks, here shown at constant phase ϕ. This observation is in line with previous (incoherent) reflectivity measurements of SAWs (Tucoulou *et al.*, 2000 ▶, 2001 ▶) and theoretical calculations (Schelokov *et al.*, 2004 ▶).

The lower inset in Fig. 4 ▶(*b*) shows that no satellite peaks are observed when the SAW is powered off, as expected. All measurements that do not involve a power scan have been performed at 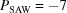
 dBm.

(3) A scan of the SAW phase ϕ in the range 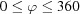
° with a step size of 5°, corresponding to a time resolution of 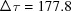
 ps. Figs. 4 ▶(*c*) and 4 ▶(*d*) show time- (phase-)dependent data for four selected values of ϕ recorded with the sCMOS and Pilatus detectors, respectively. In both cases, a strong dependence of the satellite intensity on the phase is observed, as expected. The minimum in intensity corresponds to the SAW at the instant of zero crossing, *i.e.* zero amplitude, whereas a maximum corresponds to the SAW at largest amplitude. To quantify this dependence, the intensities of the specular peak and of the (nonspecular) satellite peaks were integrated, as illustrated in Fig. 4 ▶(*d*) (red and blue dashed lines).

Fig. 4 ▶(*e*) shows the resulting traces as a function of phase ϕ. The integrated intensity of the second diffraction order is 90° out of phase with respect to the specular reflectivity. The maximum of the SAW amplitude corresponds to a maximum in the nonspecular signal, while the specular signal is decreased by a Debye–Waller-like effect. At this point it is important to note that the satellite intensity is never zero for any given value of the phase ϕ, while it is at zero when the SAW device is powered off. This suggests that the SAW contains propagating modes in addition to the desired standing wave component, most likely due to a mismatch in signal amplitude of the two IDTs. Since the propagating modes are not matched to the phase of the SR pulse, this signal component is washed out over the data accumulation interval, resulting in an offset of the ϕ scan.

## 5. Reconstruction of *h*(*x*, *t*)   

So far, we have not exploited the fact that the data were recorded under coherent illumination. In this section, we show that the one-dimensional height profile of the SAW 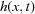 with its exact position within the KB beam envelope can be reconstructed on the basis of iterative phase reconstruction algorithms. The ϕ scan described in the previous section and shown in Fig. 4 ▶(*d*) can be used to illustrate the reconstruction of a time-dependent height profile based on the coherent reflectivity data. In a preprocessing step, the data of each diffraction pattern were integrated over a ten-pixel-wide line along 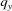 (vertical pixel position) and over 170 pixels along 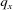 (horizontal pixel position). In this manner, the diffraction signal was averaged along 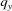 to yield a one-dimensional signal 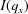 encoding just the height profile along the sound path 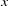. The intensity values were also corrected for the Fresnel transmission function (according to critical angle 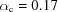
°). As explained in the section on simulations, we neglect the 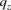 dependence of the signal 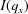, assuming a constant (mean) 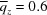
 nm^−1^ corresponding to an incident angle 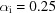
°. Each reconstruction was initialized with a random starting guess for the relative phase in the sample plane. The algorithm was run for 50 iterations, applying both modulus and real-space constraints.

For the reconstruction, we constrained the illumination wavefield to that obtained in numerical simulations (Osterhoff & Salditt, 2011 ▶). In addition, quite detailed knowledge on the KB near-field distribution is available for the GINIX endstation from recent ptychography results (Giewekemeyer *et al.*, 2013 ▶). Note that a Gaussian beam model with a width optimized to resemble the experimental far-field diffraction pattern of the KB beam was also found to yield successful reconstructions, but with inferior convergence properties. For real-space filtering, the central SAW frequency 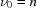 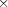 0.13 µm^−1^, 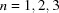, and a constant bandwidth 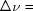 0.012 µm^1−^ was used, similar to the reconstruction of simulated data. Comparing different implementations and parameters of the bandwidth algorithm, it was found that constraining the SAW bandwidth only in the central range of illuminated surface area, while leaving the phase unconstrained in the tails of the beam, stabilized the convergence.

**Figure 5:**
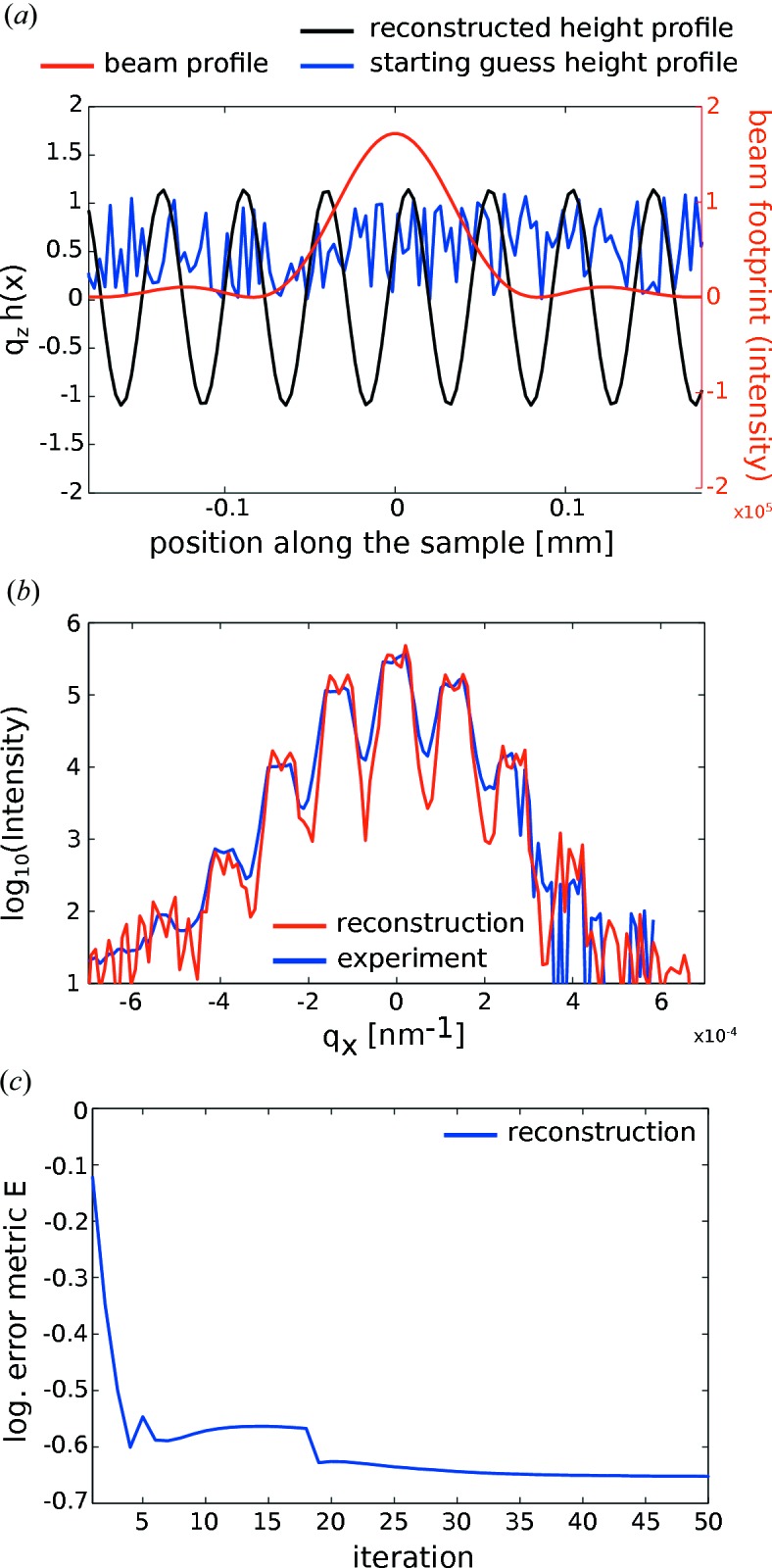
Reconstruction from experimental data, shown for one particular value of the SAW phase ϕ. (*a*) Reconstructed phase φ and beam profile (intensity) along the sample surface. The algorithm was initialized with a random starting guess (blue). The (real) beam profile was kept constant during the reconstruction process. (*b*) Experimental diffraction pattern compared with the reconstruction. Good agreement in the shape, amplitude and position of the satellite peaks is achieved. (*c*) Logarithmic error metric 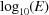.

The result of the reconstruction for one exemplary phase is shown in Fig. 5 ▶. The beam footprint as well as the reconstructed relative phase is shown in Fig. 5 ▶(*a*). The reconstructed and measured diffraction patterns in Fig. 5 ▶(*b*) agree well in amplitude and position of the satellite peaks. Convergence is achieved after 50 iterations as monitored through the logarithmic error metric 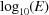, as shown in Fig. 5 ▶(*c*).

**Figure 6:**
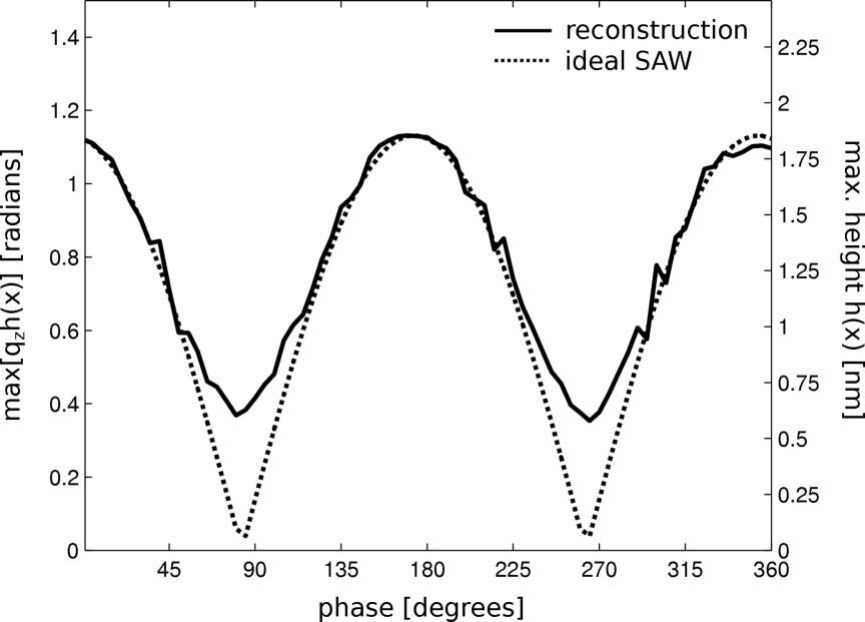
Reconstruction results. The maximum value of the height function 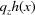 is plotted as a function of the phase angle ϕ (frequency generator to synchrotron pulse) or time. The profile agrees with the expected 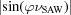 behavior, aside from an offset attributed to propagating SAW components, which is also consistent with the raw data in Fig. 4 ▶(*e*).

Central to our investigation is the reconstruction of time-resolved data. Thus, we apply the reconstruction procedure to the entire set of diffraction profiles, and the unit-less height function 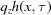 was extracted for all values of the phase ϕ. Displaying 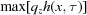 as a function of time delay τ or the phase ϕ, correspondingly, we finally get the result shown in Fig. 6 ▶. The solid curve corresponds to the reconstruction of the surface height profile for all phases ϕ. This reconstruction process was repeated with randomized initialization to give 50 single reconstruction data sets. The results after 50 iterations were found to be in very good agreement. The reconstruction corresponding to the lowest error metric is plotted in Fig. 6 ▶. One would expect a profile that is proportional to 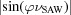, as indicated by the dotted line. This assumption cannot be confirmed by the reconstruction. As mentioned above, consistent with the raw diffraction data, the reconstruction shows no zero crossing. Since the phase stability of synchronization was well controlled during the entire experiment, this can only be explained by a significant fraction of SAW modes that propagate and do not form a standing wave. Hence such contributions can be quantified by the experimental method presented here. Let us now proceed to determine the accuracy of our reconstruction method in more detail. To estimate how accurately the amplitude of the relative phase could be found, we repeatedly reconstructed the relative phase from a single diffraction profile. On the basis of 50 different random initializations, a mean value 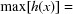 1.83 nm and standard deviation 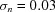
 nm were found. Next, we analyzed how stable the position of the reconstructed SAW profiles is in time. We found that the standard deviation of positional fluctuations 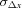 is approximately one-fifth of the SAW wavelength. This underlines the fact that the final reconstruction is properly phase stable.

## 6. Summary and outlook   

In this work we have addressed the reconstruction of time-resolved coherent diffraction data recorded in reflection geometry, as a somewhat technical step towards the goal of time-resolved coherent diffraction imaging. To this end, we have chosen surface acoustic waves (SAWs) excited on a piezoelectric substrate as a well characterized and controlled model system for aperiodic processes, which can be synchronized to the synchrotron pulses. The example shows that the height profile function of the SAW can be reconstructed with a temporal sampling of 178 ps and sub-ångström accuracy for the SAW amplitude. For the reconstruction, a dedicated algorithm was devised, based on a bandwidth constraint suitable for the current application of well controlled SAW frequency. Note that no support constraint was used. Oversampling was achieved by use of a KB focusing.

Notwithstanding the significant potential of the method for reconstruction of time-dependent interface profiles, there are several restrictions and points to note about the experimental parameters. First, 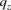 has been assumed to be constant for reconstruction purposes. This assumption is a rather major simplification and could be replaced, for example, by a more general treatment in future. Second, the beam profile has a significant role and independent quantification of the wavefront, for example by ptychography (Faulkner & Rodenburg, 2004 ▶; Rodenburg & Faulkner, 2004 ▶), including possibly ptychography in reflection mode, would be a valuable extension of this work. Third, the height profile was reconstructed as a function of only one spatial dimension. This was a choice in view of the largest possible simplification for this proof-of-concept experiment. No principle reasons stand against the feasibility of reconstruction in higher dimensions (two and three). On the contrary, significantly less *a priori* knowledge can be expected to be necessary in this case. Finally, the surface field of view in this demonstration was rather small compared to the SAW wavelength. More interesting configurations of SAW patterns in the presence of surface defects, SAW reflectors or splitters can be addressed in follow-up experiments. To this end, an adjustable beam size in combination with suitable detector pixel sizes is needed. A first step towards the illumination of larger fields of view by shifting the sample to a defocus position has already been made, but reconstruction of the data recorded in the corresponding holographic mode has to date not been achieved. This issue should also be addressed in following experiments.

However, the last argument can also be turned around. Focused coherent beams with cross sections in the range of a few hundred nanometres are ideally suited to probe functional devices with strong structural heterogenieties, for example SAW-based frequency filtering devices, by scanning the beam over the desired region of interest. Experiments in both transmission and reflection geometry are possible, but the strong phase shift induced by interface profiles leads to particularly high signal levels in reflection. Finally and more generally, we have shown that a combination of CXDI with a suitable timing scheme offers significant potential to study surfaces and interfaces a high spatial and temporal resolution.

## Figures and Tables

**Figure 7:**
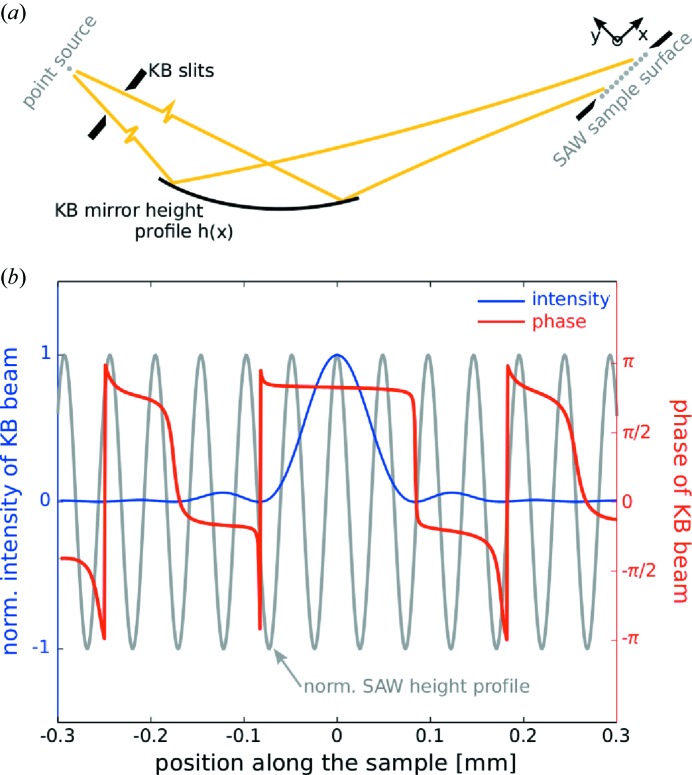
Simulated intensity and phase along the propagation direction. The simulation has been performed as detailed by Osterhoff & Salditt (2011 ▶). The normalized SAW height profile is depicted in gray for comparison. Note that the phase along the central maxima is constant in between the zeros of the intensity but jumps by a factor π at the zero crossing (to the first side lobes of the KB beam).

**Figure 8:**
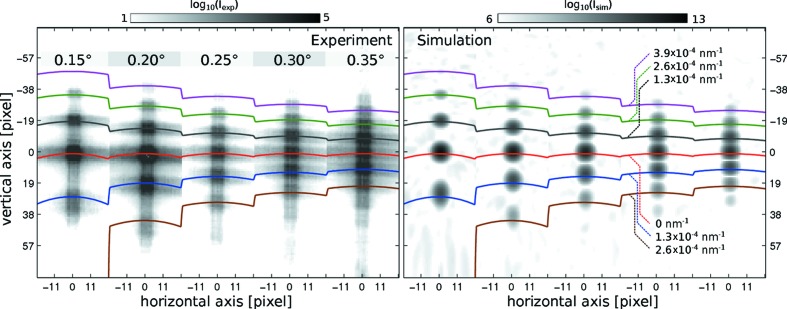
Experimental (left) and simulated (right) diffraction intensities as measured and simulated for a SAW with wavelength 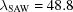
 µm and amplitude 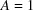
 nm, using the experimental parameters of detector distance 5.2 m and pixel size 172 µm. The incident angle is varied from 0.15 to 0.35° in 0.05° steps (left to right). 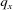 isolines with spacing 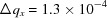
 nm^−1^ (corresponding to a real-space interval 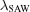) are superimposed for comparison. All satellite peaks of the simulated data are very well matched in position to the experimental satellite peaks. A Gaussian illumination profile was used in the simulation, resulting in a Gaussian lineshape of the satellites (simulated far-field pattern).

**Table 1:** Key parameters for the experiment conducted for this work

Parameter	Value	Unit
Revolution frequency 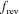	130.1	kHz
Bunchmode	40	–
Bunch frequency 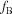	5.2	MHz
Central SAW frequency 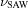	78.1	MHz
SAW wavelength 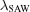	48.8	μm
Energy	13.8	keV
Focal spot	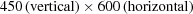	nm
Beam divergence	1.5	mrad
Sample–detector distance	5.2	m
Pixel size	172/6.75	μm
Angle of incidence	Minimum 0.15, maximum 0.35	°
SAW power 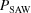	Minimum −21, maximum −7	dBm

## Appendix A Additional numerical simulations

In this appendix we give further information on numerical simulations of the illumination function, corresponding to the KB mirror system used at GINIX. The simulation results as shown in Fig. 7 ▶ have been performed using the approach presented by Osterhoff & Salditt (2011 ▶) and show that the phasefront in the KB focal plane is sufficiently flat [see central region in Fig. 7 ▶(*b*)], so that the reality constraint for the illumination function is well justified.

Next, we have included a figure comparing the two-dimensional experimental intensity distribution and its changes as a function of  with the corresponding forward simulations (Fig. 8 ▶).

Here, a Gaussian model has been assumed for the illumination profile. The results show that the patterns and in particular positions of the satellites are in excellent agreement. An extension of the present reconstruction results obtained from a singe  data set could take into account a range of  and would possibly allow for a reconstruction approach in three dimensions, provided that suitable regridding of the data is performed.
